# Evidence for the Involvement of Lipid Rafts and Plasma Membrane Sphingolipid Hydrolases in *Pseudomonas aeruginosa* Infection of Cystic Fibrosis Bronchial Epithelial Cells

**DOI:** 10.1155/2017/1730245

**Published:** 2017-12-03

**Authors:** Domitilla Schiumarini, Nicoletta Loberto, Giulia Mancini, Rosaria Bassi, Paola Giussani, Elena Chiricozzi, Maura Samarani, Silvia Munari, Anna Tamanini, Giulio Cabrini, Giuseppe Lippi, Maria Cristina Dechecchi, Sandro Sonnino, Massimo Aureli

**Affiliations:** ^1^Dipartimento di Biotecnologie Mediche e Medicina Traslazionale, Università degli Studi di Milano, Milano, Italy; ^2^Laboratorio di Patologia Molecolare-Laboratorio di Chimica Clinica ed Ematologia, Dipartimento di Patologia e Diagnostica, Azienda Ospedaliera Universitaria Integrata di Verona, Ospedale Civile Maggiore, Verona, Italy; ^3^Sezione di Biochimica Clinica, Università degli Studi di Verona, Verona, Italy

## Abstract

Cystic fibrosis (CF) is the most common autosomal genetic recessive disease caused by mutations of gene encoding for the cystic fibrosis transmembrane conductance regulator. Patients with CF display a wide spectrum of symptoms, the most severe being chronic lung infection and inflammation, which lead to onset of cystic fibrosis lung disease. Several studies indicate that sphingolipids play a regulatory role in airway inflammation. The inhibition and downregulation of GBA2, the enzyme catabolizing glucosylceramide to ceramide, are associated with a significant reduction of IL-8 production in CF bronchial epithelial cells. Herein, we demonstrate that GBA2 plays a role in the proinflammatory state characterizing CF cells. We also report for the first time that *Pseudomonas aeruginosa* infection causes a recruitment of plasma membrane-associated glycosphingolipid hydrolases into lipid rafts of CuFi-1-infected cells. This reorganization of cell membrane may be responsible for activation of a signaling cascade, culminating in aberrant inflammatory response in CF bronchial epithelial cells upon bacterial infection. Taken together, the presented data further support the role of sphingolipids and their metabolic enzymes in controlling the inflammatory response in CF.

## 1. Introduction

Cystic fibrosis (CF) is the most common autosomal genetic recessive disease in Caucasian population, affecting approximately 1 in 2500–4000 newborns [1]. CF is caused by mutations of the gene encoding for cystic fibrosis transmembrane conductance regulator (CFTR).

The main phenotype of CF is characterized by accumulation of viscous mucus at the epithelial surface of different organs such as the lungs, pancreas, gut, and testes, often resulting in inflammation and organ failure. CF patients usually die prematurely due to onset of cystic fibrosis lung disease, originating from chronic lung infection and inflammation [1]. The hallmarks of increased inflammation in CF are high levels of interleukin (IL)-1*β*, IL-6, IL-8, and tumor necrosis factor-*α* (TNF*α*).

IL-8 is abundantly expressed at sites of chronic infection and its expression seems correlated with generation of neutrophil- (PMN-) rich exudates in the lungs of CF patients [2–5]. The IL-8 cascade may hence represent a key therapeutic target for mitigating CF lung inflammation. Several factors contribute to the severity of CF inflammation. Among these, an increasing number of studies highlight that sphingolipids (SLs) play an important regulatory role in CF pulmonary infection and inflammation [6–14].

SLs are predominantly present in the external leaflet of the plasma membrane. These amphipathic molecules are characterized by a hydrophobic moiety (ceramide), inserted into the cellular lipid bilayer, and a hydrophilic group of different complexity protruding toward extracellular environment [15].

SLs are not merely structural components of biological membranes, but play other important roles in regulation of cell physiology [16]. At the plasma membrane (PM), SLs, together with cholesterol, saturated phospholipids, and a specific pool of proteins, form macromolecular complexes that are conventionally defined lipid rafts [17, 18].

Several studies have depicted membrane rafts as dynamic nanoscale domains playing an important role in cell signal transduction [19]. Interestingly, modifications of lipid raft SL composition are associated with many cellular processes, such as neuronal differentiation and senescence, insulin resistance, and inflammatory response to bacterial infections [8, 20, 21].

Several processes concur to determine the PM SL pattern and content, that is, neobiosynthesis in the endoplasmic reticulum and Golgi apparatus and catabolism in lysosomes. Recent reports also suggest that the enzymes involved in the SL metabolism are also present within the cell PM [22].

In particular, the presence of different hydrolases involved in the in situ reorganization of the PM SL composition has been described at the cell surface of several cell lines. Sphingomyelinases (SMase), *β*-galactosidase (*β*-Gal), *β*-hexosaminidase (*β*-Hex), sialidase Neu3, *β*-glucocerebrosidase GBA1, and the nonlysosomal *β*-glucosylceramidase GBA2 have already been identified [23, 24]. These PM-associated hydrolases seem to play an active role in the inflammatory response of CF bronchial epithelial cells subjected to *P. aeruginosa* infection, although a direct correlation has not been reported so far [25].

Genetic silencing and pharmacological inhibition of acid sphingomyelinase reduce Cer levels, thus resulting in decreased inflammatory response to *P. aeruginosa* infection [26].

On the other hand, the catabolism of complex glycosphingolipids (GSL) at PM level seems to play an important role in the proinflammatory state of CF and in inflammation caused by *P. aeruginosa* infection [25].

Of considerable interest is the nonlysosomal beta-glucosylceramidase GBA2, an enzyme involved in the final hydrolysis of glucosylceramide (GlcCer) to ceramide at the cell surface. Recent data show that inhibition of GBA2 with miglustat reduces the inflammatory response in CF bronchial epithelial cells infected by *P. aeruginosa* [25].

Moreover, GBA2 knockdown in noninfected cells induces a reduction of IL-8 basal level and, therefore, of the intrinsic proinflammatory state [25].

Nevertheless, the mechanistic link between involvement of GBA2 or other PM-associated hydrolases and inflammatory response in CF is unknown. We herein report data supporting the role of GBA2 in the inflammatory response to *P. aeruginosa* infection. We also describe that *P. aeruginosa* infection causes a reorganization of the lipid rafts isolated from CF bronchial epithelial cells.

## 2. Materials and Methods

### 2.1. Cell Culture

The CuFi-1 and NuLi-1 cell lines were a generous gift of A. Klingelhuts, Pkarp, and J. Zbaner, University of Iowa, Iowa City [27], and were cultured as previously described [25]. Briefly, the cells were cultured as monolayer in a humidified atmosphere at 37°C and 5% CO_2_ in flasks precoated with collagen (collagen IV from human placenta, Sigma-Aldrich) in bronchial epithelial growth medium (BEGM by Lonza; singleQuot Kit Lonza).

### 2.2. Generation of CuFi-1 and NuLi-1 Cell Lines Overexpressing GBA2-GFP

CuFi-1 and NuLi-1 cell lines overexpressing GBA2 tagged with GFP were generated by 3rd generation lentiviral particles carrying a lentiviral vector coding for GBA2-GFP fusion protein (pLenti-GBA2-GFP). A lentiviral vector containing only GFP (pLenty-GFP) was used to generate mock cells. Viral particles were generated by transfecting 293FT cells with 3 *μ*g of pLenti-GBA2-GFP or pLenti-GFP vectors and 9 *μ*g of packaging vector mix (coding for Gag, Pol Tat, Rev, and VSVG), using Lipofectamine™ 2000 Reagent (Invitrogen).

Supernatant media were collected 48 h after transfection, filtered through a 0.4 *μ*m membrane, and used to transduce cells according to manufactures instructions.

CuFi-1 and NuLi-1 cells were infected with 1 ml of each lentivirus stock. After 1 day, the medium was replaced with fresh medium containing the specific antibiotic to select positive clones. To obtain constitutive clones, transduced cells were identified with 5 *μ*g/ml of puromycin. After 3 weeks of selection, colonies were separated and expanded. The cells were cultured as monolayer in a humidified atmosphere at 37°C and 5% CO_2_ in flasks, precoated with collagen (collagen IV from human placenta, Sigma-Aldrich) and using bronchial epithelial growth medium (BEGM by Lonza; singleQuot Kit Lonza) in the presence of 1 *μ*g/ml of puromycin.

Clones obtained were morphologically analyzed for GBA2 tagged with GFP or GFP expression by confocal microscopy after fixing cells with paraformaldehyde (2% in PBS) for 20 min. The expression of GBA2 mRNA was measured by real-time qRT-PCR as described [28, 29].

### 2.3. Bacterial Strain

For infection experiments, heat-killed *P. aeruginosa* strain PAO1, kindly provided by A. Prince (Columbia University, New York), was used.

PAO1 was grown in a Trypticase soy broth (TSB) or agar (TSA), as described by Dechecchi and colleagues [30]. The organism was killed by heating to 65°C for 30 min [25].

### 2.4. Bacterial Infection and Evaluation of IL-8 Secretion

Cells were infected for 4 h with PAO1 (100 CFU/cells). Quantitative measurement of IL-8 protein release in the cell medium was evaluated using human IL-8 instant ELISA kit (Bender MedSystems, Wien, Austria). The expression of IL-8 mRNA was measured by real-time qRT-PCR as previously described [29].

### 2.5. Cell Sphingolipid Labelling with [1-^3^H]-Sphingosine

[1-^3^H]-sphingosine was administered as tracer at nonbioactive concentration for 2 h (pulse), to allow steady-state metabolic labelling of all cell SLs [31]. Briefly, [1-^3^H]-sphingosine dissolved in methanol was transferred into a sterile glass tube, dried under a nitrogen stream and then solubilized in an appropriate volume of a prewarmed (37°C) medium (BEGM) to obtain a final concentration of 0.3 nM.

The correct solubilisation was verified by measuring the radioactivity associated with an aliquot of a medium by beta-counter (PerkinElmer). After 2 h of incubation (pulse), the medium was removed and the cells were incubated for 48 h (chase) in a fresh culture medium without radioactive sphingosine. After chase, cells were collected and used to perform analysis of radioactive lipids or detergent-resistant membrane (DRM) preparation.

### 2.6. Isolation of Detergent-Resistant Membrane Fractions

DRM were prepared by ultracentrifugation on discontinuous sucrose gradient of cells subjected to homogenization with 1% Triton X-100, as previously described [32]. Briefly, cells were mechanically harvested in PBS and centrifuged at 270 ×g for 10 min. Cell pellet was lysed in 1.2 ml of 1% Triton X-100 in TNEV buffer (TrisHCl 10 mM, NaCl 150 mM, EDTA 5 mM, pH 7.5) in the presence of 1 mM Na_3_VO_4_, 1 mM PMSF, and 75 mU/ml aprotinin and homogenized with tight Dounce.

Cell lysate (2 mg of cell protein/ml) was centrifuged for 5 min at 1300 ×g to remove nuclei and cellular debris, to obtain postnuclear supernatant (PNS).

A volume of 1 ml of PNS was mixed with 1 ml of 85% sucrose (*w*/*v*) in TNEV buffer containing 1 mM Na_3_VO_4_, placed at the bottom of a discontinuous sucrose gradient (30% and 5%), and centrifuged for 17 h at 200,000 ×g at 4°C.

After ultracentrifugation, 11 fractions were collected. The light-scattering band, corresponding to the DRM fraction, was located at the interface between 5% and 30% sucrose corresponding to fraction 4 or 5. Equal amounts of low-density fractions (4 and 5) were mixed to obtain DRM fraction, whereas equal amounts of high-density fractions (10 and 11) were mixed to obtain the HD fraction used for lipid analyses and hydrolase assays. The entire procedure was performed at 0–4°C on ice immersion.

### 2.7. Sphingolipid Analysis

Cell lysates or DRM and HD fractions were dialyzed, lyophilized, and subjected to lipid extraction and SL analyses. Total lipids were extracted with CHCl_3_/CH_3_OH/H_2_O 20 : 10 : 1 by vol, followed by a second extraction with CHCl_3_/CH_3_OH 2 : 1 by vol. The total lipid extracts were subjected to a two-phase partitioning by adding 20% water to lipid extract. The radioactivity associated with total lipid extract, aqueous, and organic phases was evaluated by liquid scintillation, using a beta-counter system (PerkinElmer). Organic phases were subjected to alkali treatment to remove glycerophospholipids, as described elsewhere [33].

[^3^H]SLs of total extracts and organic phases were separated by high-performance thin layer chromatography (HPTLC), using the solvent system CHCl_3_/CH_3_OH/H_2_O 110 : 40 : 6 by vol, and those of aqueous phases with CHCl_3_/CH_3_OH/0.2% aqueous CaCl_2_, 50 : 42 : 11 by vol. [^3^H]-SLs were identified by digital autoradiography using the ^T^Racer system (Biospace Lab) and quantified with M3vision software. The lipid identification was performed using purified radioactive standards [33].

### 2.8. Enzymatic Activity Associated with the Cell Plasma Membrane

PM-associated activities of *β*-galactosidase (*β*-Gal), *β*-glucosidase GBA1, *β*-glucosidase GBA2, and *β*-hexosaminidase (*β*-Hex) were assessed in living cells, plated in 96-well microplates at a density of 20,000 cells/well, by a high-throughput cell live-based assay, as previously described [34, 35].

In order to distinguish between GBA1 and GBA2 activity, cells were preincubated for 30 min at room temperature in DMEM-F12 containing 5 nM AMP-dNM (adamantane-pentyl-dNM; N-(5-adamantane-1-yl-methoxy-pentyl) deoxynojirimycin) a specific inhibitor of GBA2 or 1 mM CBE (conduritol-B-epoxide) (Sigma) a specific inhibitor of GBA1 [36].

*β*-Gal, *β*-Hex, and *β*-Glc activities were assayed using the artificial substrates 4-methylumbelliferyl-*β*-D-galactopyranoside (MUB-Gal), 4-methylumbelliferyl-*N*-acetyl-*β*-D-glucosaminide (MUG), and 4-methylumbelliferyl-*β*-D-glucopyranoside (MUB-Glc) solubilized in DMEM-F12 without phenol red at pH 6, at final concentrations of 250 *μ*M, 2 mM, and 6 mM, respectively.

Aliquots of medium (10 *μ*l) were analyzed at different time points with a fluorimeter in a microplate reader (Victor, PerkinElmer) (MUB: *λ*ex: 355 nm/*λ*em: 460 nm), after adding 190 *μ*l of 0.25 M glycine, pH 10.7.

Standard free MUB was used to construct a calibration curve and to quantify substrate hydrolysis. The enzymatic activity was expressed as pmoles of product/10^6^ cells/h.

The experimental design was made considering all the controls in terms of both cell line used and internal control for the experimental procedures. In particular, this method is based on the observation that the fluorigenic substrates commonly used for the *in vitro* assay of glycohydrolase activities are not taken up by living cells. To be sure that the substrate hydrolysis occurs only upon the activity of PM enzymes, a series of control were performed. In the used experimental condition, we did not observe any fluorescence associated with cells confirming that the substrates were not able to cross the cell membrane. Moreover, we verified that the artificial substrates did not undergo spontaneous hydrolysis or hydrolysis driven by secreted enzymes. Thus, their hydrolysis under these experimental conditions is due exclusively to the PM-associated enzymatic activities.

### 2.9. Enzymatic Activities Associated with Cell Lysate and Sucrose Gradient Fractions

The enzymatic activities of *β*-galactosidase (*β*-Gal), *β*-glucosidase GBA1, *β*-glucosidase GBA2, *β*-hexosaminidase (*β*-Hex), and sphingomyelinases (SMase) were determined in the total cell lysates using fluorigenic substrates, as previously described with few modifications [37, 38].

Briefly, cells were washed twice with PBS, harvested, and suspended in water in the presence of a protease inhibitor cocktail (Sigma-Aldrich). Total cell proteins were evaluated by DC™ protein assay (Bio-Rad), performed according to manufacturer instructions.

Equal amounts of cell proteins were transferred into a 96-well microplate and the assay was performed 3-fold in replicate. MUB-Glc was solubilized in McIlvaine buffer (pH 6) at concentration of 6 mM. MUB-Gal, MUG, and H-MUB-PC (used for SMase assay) were solubilized in McIlvaine buffer (pH 5.2) at concentrations of 500 *μ*M, 1 mM, and 500 *μ*M, respectively.

In order to distinguish between GBA1 and GBA2 activity, cells were preincubated for 30 min at room temperature in McIlvaine buffer (pH 6) with 5 nM AMP-dNM (adamantane-pentyl-dNM; N-(5-adamantane-1-yl-methoxy-pentyl) deoxynojirimycin) a specific inhibitor of GBA2 or 1 mM CBE (conduritol-B-epoxide) (Sigma) a specific inhibitor of GBA1.

The reaction mixtures were incubated at 37°C under gentle shaking. After different times of incubation, 10 *μ*l of the reaction mixtures was transferred to a 96-black-well microplate adding 190 *μ*l of glycine (0.25 M, pH 10.7). In the case of H-MUB-PC, 0.3% of Triton X-100 were added to glycine solution. Fluorescence was detected at different time points with a Victor microplate reader (PerkinElmer).

Standard free MUB and H-MUB were used to construct calibration curves for quantifying substrate hydrolysis. The enzymatic activity was expressed as pmoles of product/mg protein/h. The enzymatic activity associated with sucrose gradient fractions was expressed as pmoles of product/volume of fraction/h. Due to the presence of Triton X-100 detergent, we could not detect GBA2 activity associated with gradient fractions.

### 2.10. Statistical Analysis

All the experiments were performed in triplicate otherwise differently indicated, and statistical significance was determined by Student-Neumann-Keuls post hoc test (comparison between two groups) and by one-way or two-way ANOVA (followed by Turkey or Dunnett Neueman-Keuls on Bonferroni post test) for more than two groups, with statistical significance set at *p* < 0.05, using GraphPad Prism 5.

## 3. Results

### 3.1. PM-Associated Glycohydrolases Are Involved in Controlling the Proinflammatory State in CF

We previously reported an association between GBA2 silencing and reduction of IL-8 expression in CF bronchial epithelial cells [25]. To date, it is accepted that the accumulation of Cer correlates with the intrinsic proinflammatory state of CF and the age-dependent hypersusceptibility to infections [8]. Interestingly, this inflammatory state is a feature of CuFi-1 cells, a widely used CF bronchial epithelial cell model that shows a higher basal IL-8 secretion compared to non-CF bronchial epithelial cells, NuLi-1.

**Figure 1:**
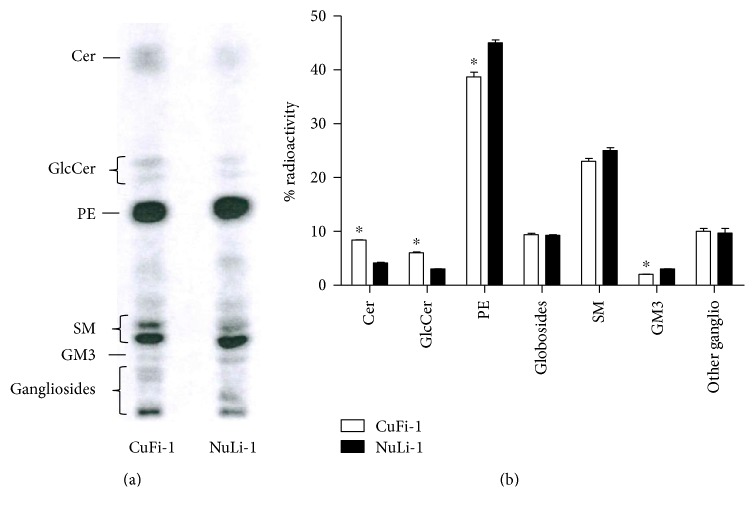
Sphingolipid composition of CuFi-1 and NuLi-1 cells. (a) HPTLC of radioactive sphingolipids obtained from total lipid extract of CuFi-1 and NuLi-1 cells. (b) Semiquantitative graph of sphingolipids species. The sphingolipids are represented as percentage of radioactivity. Cer: ceramide; GlcCer: glucosylceramide; PE: phosphatidylethanolamine; SM: sphingomyelin. The sphingolipid composition of CuFi-1 and NuLi-1 cells was evaluated performing three different experiments and for each experiment, one HPTLC was performed. ^∗^*p* < 0.05 versus NuLi-1.

The analysis of SL composition of both cell lines (Figure 1) showed that CuFi-1 cells have an increased content of Cer and GlcCer, followed by a slight reduction of ganglioside GM3 compared to NuLi-1 cells.

**Figure 2:**
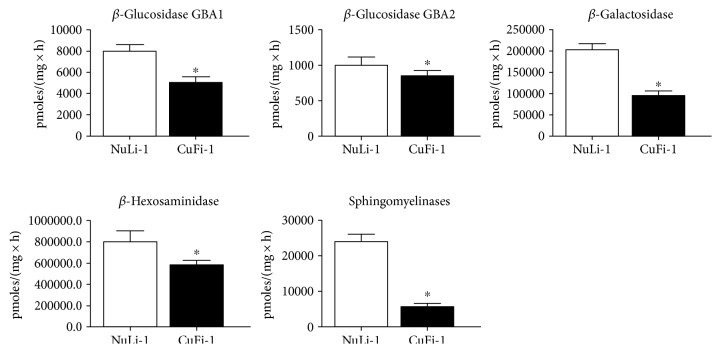
Hydrolases activity associated with total cell lysate of CuFi-1 and NuLi-1 cells. The measurements of the hydrolases activity were conducted on cell lysates using an in vitro enzymatic assay based on artificial fluorigenic substrates. The enzymatic activity is expressed as pmoles of product on mg of proteins per hour. ^∗^*p* < 0.001 versus NuLi-1.

**Figure 3:**
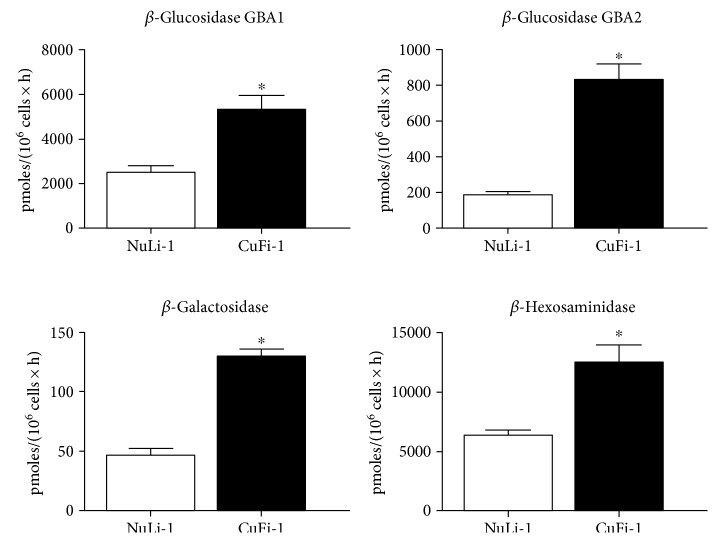
Hydrolase activities associated with cell plasma membranes of CuFi-1 and NuLi-1 cells. The measurements of the plasma membrane hydrolase activity were conducted on living cells, using artificial fluorigenic substrates. The enzymatic activity is expressed as pmoles of product, on 10^6^ cells per hour. ^∗^*p* < 0.005 versus NuLi-1.

We measured the activity of the main hydrolases involved in SL catabolism, observing that the activity of all enzymes tested was higher in NuLi-1 compared to CuFi-1 cells (Figure 2). Interestingly, opposite results were obtained by assay of hydrolases activity measured at PM level.  Figure 3 shows that CuFi-1 cells have a twofold increase in the activity of hydrolases present at cell PM such as GBA1, *β*-Gal, and *β*-Hex, compared to NuLi-1 cells. Notably, the activity of GBA2 was 4-fold higher in CuFi-1 compared to NuLi-1 cells. This data is consistent with our previous results [25] on the role of GBA2 in inflammatory response. To further support these findings, we stably overexpressed GBA2 in CuFi-1 cells as GFP fusion protein. As control cells, we generated mock cell lines overexpressing GFP.

**Figure 4:**
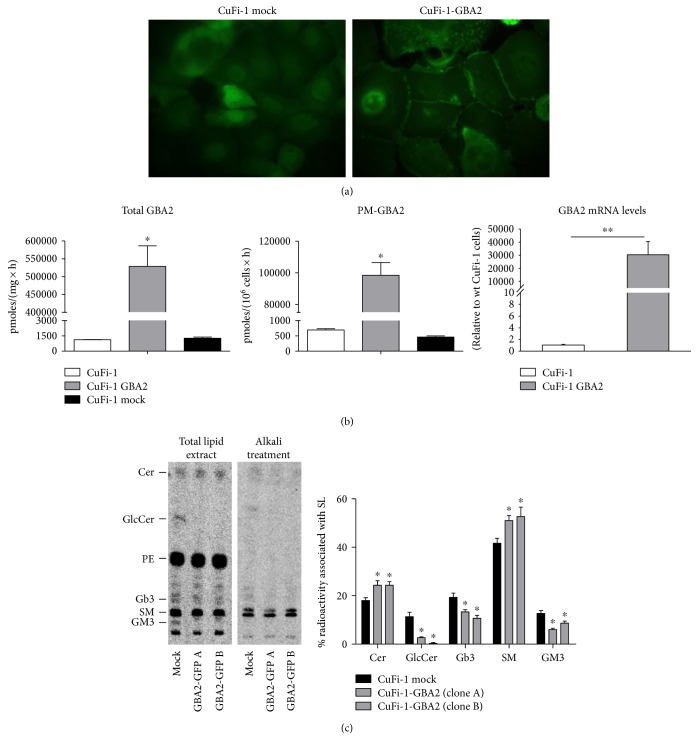
CuFi-1 cells overexpressing GBA2 tagged with GFP. (a) Confocal images of CuFi-1 cells overexpressing GFP (CuFi-1 mock) and overexpressing GBA2-tagged GFP (CuFi-1-GBA2). (b) Total and plasma membrane enzymatic activity of GBA2 measured in parental CuFi-1 cells and CuFi-1-overexpressing GBA2 and mock cells. The measurement of the total GBA2 enzymatic activity was conducted on cell lysate and the activity expressed as pmoles of product on mg of proteins per hour. The measurement of the plasma membrane enzymatic activity of GBA2 was conducted on living cells and expressed as pmoles of product on 10^6^ cells per hour. ^∗^*p* < 0.0001 versus mock. On the right mRNA expression of GBA2 in CuFi-1 and GBA2-overexpressing cells evaluated by qRT-PCR, data are expressed as fold increase with respect to control cells. ^∗∗^*p* < 0.001 versus CuFi-1. (c) Radioactive sphingolipid composition of CuFi-1-overexpressing GBA2 compared to mock cells. Cell sphingolipids were metabolic labeled at the steady state using [1-^3^H]-sphingosine as indicated in Materials and Methods. Left: representative digital autoradiography of radioactive lipids of two different clones of CuFi-1-overexpressing GBA2 and in one clone of mock cells. Right: semiquantitative graph of sphingolipid species. Data are reported as percentage of radioactivity associated with sphingolipids. Cer: ceramide; GlcCer: glucosylceramide; PE: phosphatidylethanolamine; Gb3: globotriaosylceramide; SM: sphingomyelin. ^∗^*p* < 0.05 versus CuFi-1 mock cells

After 3 weeks, several clones were morphologically analyzed by confocal microscopy, to detect GFP-tagged GBA2. As shown in Figure 4(a), GBA2-overexpressing cells (CuFi-1-GBA2) are characterized by a clear signal at the cell surface compared to mock cells, although a diffuse intracellular fluorescence was observed.

The activity of GBA2 in mock (CuFi-1 mock) and in GBA2-overexpressing (CuFi-1-GBA2) cells was measured both in total cell lysate and at the PM. As shown in Figure 4(b), GBA2-overexpressing cells are characterized by increased GBA2 activity, 300-fold and 130-fold higher in total cell lysate and cell PM, respectively.

The effect of GBA2 overexpression on the SL pattern was analyzed after metabolic labeling at the steady state, with the radioactive precursor [1-^3^H]-sphingosine. GBA2-overexpressing CuFi-1 cells are characterized by a strong decrease of GSL such as GM3 and GlcCer, followed by increased Cer and SM content (Figure 4(c)). Interestingly, higher GBA2 activity induces a forced catabolism of GM3 and GlcCer to ceramide, which can be converted to SM, as previously suggested [39].

Several lines of evidence suggest that cells react to activity variations of one of the enzymes of SL metabolism by modifying the function of other enzymes involved in the same catabolic pathway [28]. For this reason, we measured the activity of the main cell hydrolases.

**Figure 5:**
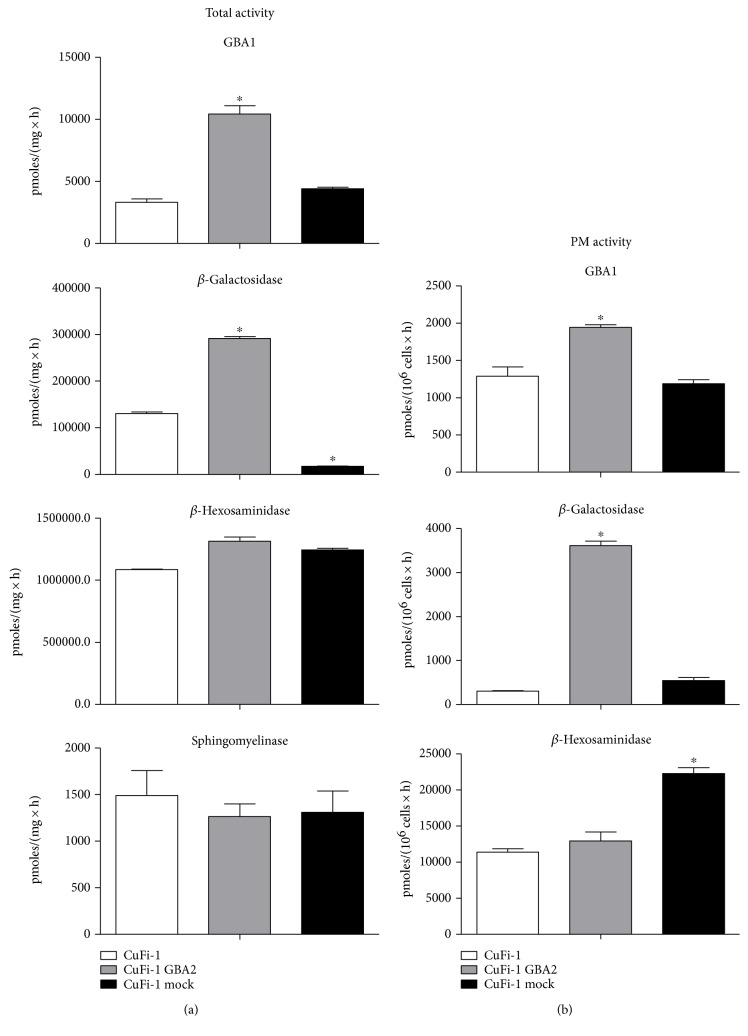
Effect of GBA2 overexpression on the enzymatic activity of the main sphingolipid hydrolases. (a) Total cell associated hydrolases activity of parental CuFi-1, CuFi-1-GBA2, and mock cells. The measurements of the hydrolases activity were conducted on cell lysate and expressed as pmoles of product/mg of proteins per hour. (b) Plasma membrane hydrolase activity in parental CuFi-1, CuFi-1-GBA2, and mock cells. The measurements of the plasma membrane hydrolase activity were conducted on living cells and expressed as pmoles of product/10^6^ cells per hour. ^∗^*p* < 0.0001 versus CuFi-1.

Our results show that overexpression of GBA2 in CuFi-1 cells causes an increase of GBA1 and *β*-Gal activity in total cell lysate. In mock and parental CuFi-1 cells, GBA1 activity is 3857 ± 635 pmoles/mg/h, increasing to 10,408 ± 690 pmoles/mg/h in CuFi-1-GBA2, whereas *β*-Gal increased in CuFi-1-GBA2 compared to CuFi-1 or mock cells (Figure 5). We also evaluated the activity of GBA1, *β*-Gal, and *β*-Hex at the PM of living mock- and GBA2-overexpressing cells. As shown in Figure 5, a slight increase of GBA1 was observed compared to mock cells, whereas the PM-associated *β*-Gal increased more than 50-fold in CuFi-1-GBA2 cells compared to mock or CuFi-1 cells.

Significant differences in total *β*-Gal and PM-associated *β*-Hex were found between CuFi-1 and mock cells, probably caused by the different passages in culture due to the transfection process. Unfortunately, the CuFi-1 cell line is characterized by an undetectable SMase activity at the PM level (data not shown).

**Figure 6:**
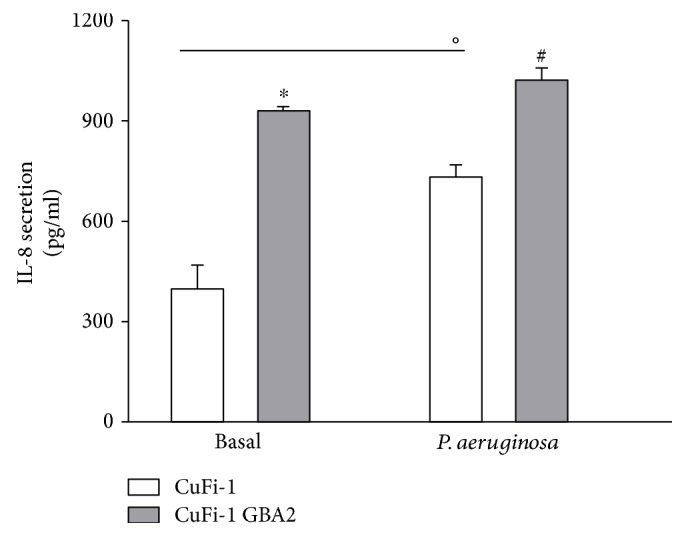
Effect of GBA2 overexpression on inflammatory response of CuFi-1 cells. Quantitative measurement of IL-8 protein release in the cell medium of CuFi-1 and CuFi-1-GBA2 cells infected or not with PAO1 for 4 h (100 CFU/cell). Data are mean ± SEM of three independent experiments performed in duplicate. Comparison between groups were done by Student's *t*-test. ^∗^*p* < 0.0001 versus CuFi-1 cells, °*p* < 0.01 versus CuFi-1 cells versus CuFi-1 cells infected, ^#^*p* < 0.0001 versus CuFi-1 cells infected.

Since the silencing of GBA2 causes a reduction of IL-8 in the proinflammatory state and in the inflammatory response to *P. aeruginosa*, we also investigated the effect of GBA2 overexpression by measuring IL-8 protein secretion in GBA2-overexpressing CuFi-1 cells compared to CuFi-1 cells.  Figure 6 shows that GBA2-overexpressing cells are characterized by a significant increase of IL-8 secretion in basal condition as well as in response to *P. aeruginosa* infection. We analyzed the effect of GBA2 overexpression in terms of IL-8 production also in NuLi-1 cells. We evaluated the IL-8 production in the basal status of NuLi-1 cells, as well as, after *Pseudomonas aeruginosa* infection.

**Figure 7:**
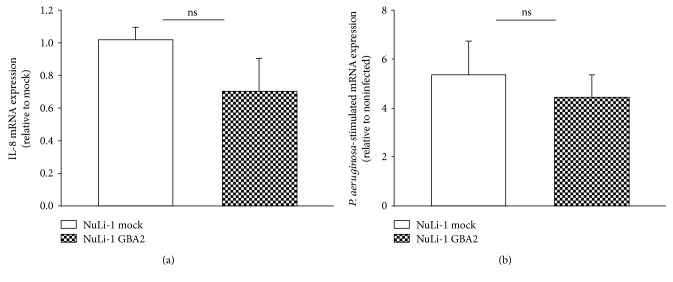
Effect of GBA2 overexpression on inflammatory response of NuLi-1 cells. (a) IL-8 mRNA expression at basal level of NuLi-1-GBA2 cells versus NuLi-1 mock cells. (b) IL-8 mRNA expression after *Pseudomonas aeruginosa* infection in NuLi-1-GBA2 cells versus NuLi-1 mock cells.

As shown in Figure 7, the results obtained indicate that the basal expression of IL-8 of NuLi-GBA2-overexpressing cells is not significantly different compared to basal IL-8 of NuLi-mock cells. This suggests that the overexpression of GBA2 in non-CF cells does not affect the basal expression of IL-8. No effect was found also in NuLi-GBA2 subjected to *P. aeruginosa* infection.

These data further support the role of GBA2 in the aberrant inflammatory state occurring in CF bronchial epithelial cells.

### 3.2. *P. aeruginosa* Infection Causes a Reorganization in the DRM Isolated from CF Bronchial Epithelial Cells

We previously showed that *P. aeruginosa* triggers significant changes in SL composition, generating an increase in Cer content [25]. As suggested by others, these alterations seem attributable to activation of SL catabolic pathways [10, 40].

We hence investigated the effect of *P. aeruginosa* infection on enzymatic activity of SMase and on the main glycohydrolases involved in regulation of Cer content (i.e., GBA1, GBA2, *β*-Gal, and *β*-Hex) in CuFi-1 cells.

**Figure 8:**
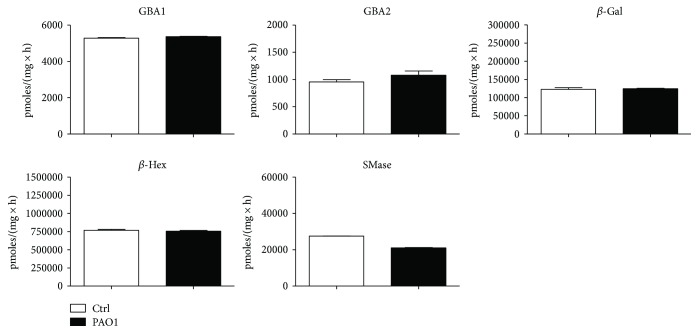
Effect of *P. aeruginosa* infection on the activity of total cell hydrolases of CuFi-1 cells. The measurements of the hydrolases activity were conducted on cell lysate by an in vitro enzymatic assay using artificial fluorigenic substrates. Before the assay, cells were infected or not for 4 hours with PAO1. The enzymatic activities are expressed as pmoles of product/mg of proteins per hour.

We first infected CuFi-1 cells with heat-killed *P. aeruginosa* strain PAO1 for 4 h and then measured hydrolases activity in total cell lysate. As shown in Figure 8, no significant difference was observed in the activity of total cell hydrolases between infected and noninfected CuFi-1 cells.

**Figure 9:**
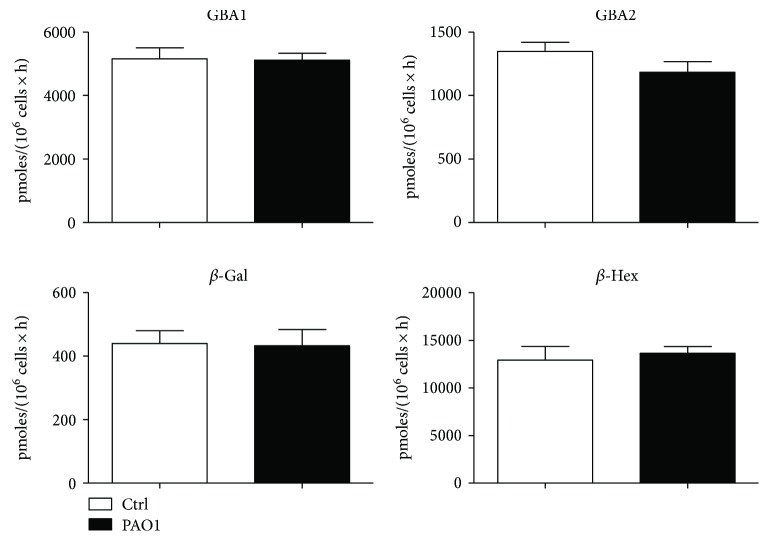
Effect of *P. aeruginosa* infection on plasma membrane hydrolases of CuFi-1 cells. The measurements of the plasma membrane hydrolase activity were conducted on living cells, using artificial fluorigenic substrates. Before the assay, cells were infected or not for 4 hours with PAO1. The enzymatic activities are expressed as pmoles of product/10^6^ per hour.

We then quantified GBA1, GBA2, *β*-Gal, and *β*-Hex activities at cell surface of CuFi-1 cells under identical experimental conditions. Neither in this case we could find significant differences between infected and noninfected CuFi-1 cells (Figure 9).

Notably, the assay used for measuring hydrolase activity at PM level allows measuring the enzymatic activity of the entire cell surface, but does not actually reflect the activity of a specific PM area.

SLs form specific membrane domains within PM, which are called lipid rafts, and are involved in activation and regulation of different intracellular pathways. In order to study the involvement of these membrane domains in PAO1 inflammatory response, we labeled cell SLs at the steady state with [1-^3^H]-sphingosine. We isolated lipid rafts from infected and noninfected cells as the detergent-resistant membrane (DRM), according to the procedure thoughtfully described in “Materials and Methods”.

For isolation of DRM fraction, the same amount of cell proteins from infected or noninfected cells, lysed with 1% Triton X-100, was loaded on a discontinuous sucrose gradient. After ultracentrifugation, eleven fractions were collected and the radioactivity associated with each of them was then assayed.

As expected, an enrichment in radioactivity was observed in low-density fractions 4 and 5, thus suggesting SL enrichment. The remaining radioactivity was present in high-density (HD) fractions.

**Figure 10:**
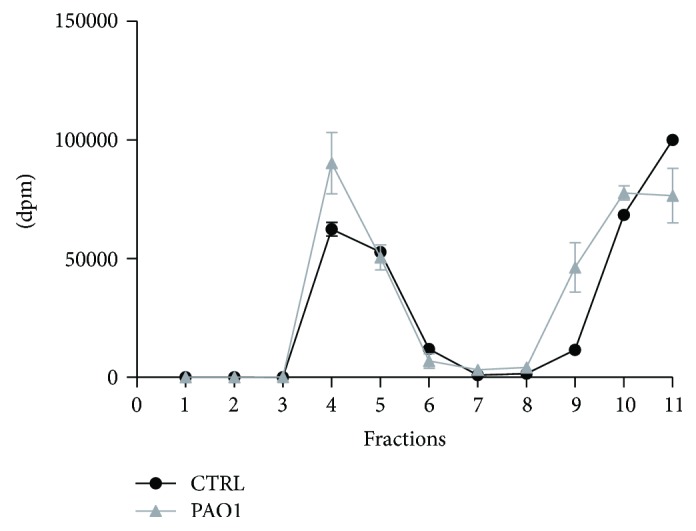
Radioactivity distribution within gradient fractions of CuFi-1 cells infected or not with *P. aeruginosa*. Cell sphingolipids were labeled at the steady state with [1-^3^H]-sphingosine. We isolated the lipid rafts from infected and noninfected cells as the detergent-resistant membrane (DRM) following the procedure described in details in “Materials and Methods”. After ultracentrifugation, we collected eleven fractions and we measured the radioactivity associated to each of them.

Interestingly, PAO1 infection causes a 20% increase of radioactivity in the DRM of infected CuFi-1 cells (Figure 10).

**Figure 11:**
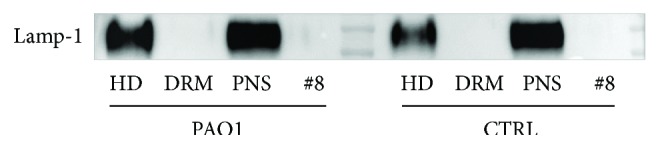
Immunoblotting against the lysosomal marker Lamp-1. Immunoblotting analyses against Lamp-1 performed on postnuclear supernatant (PNS), detergent-resistant membrane (DRM) fraction, fraction number 8 (#8), and high-density (HD) fraction from CuFi infected or not with *P. aeruginosa.*

In order to evaluate possible lysosomal contaminations, we performed an analysis of a lysosomal marker on gradient fractions. We performed an immunoblotting using an antibody against the lysosomal marker Lamp-1 on DRM and HD fractions. As reported in Figure 11, no signals are detectable in DRM fraction, whereas a marked spot is associated with HD and PNS fraction.

**Figure 12:**
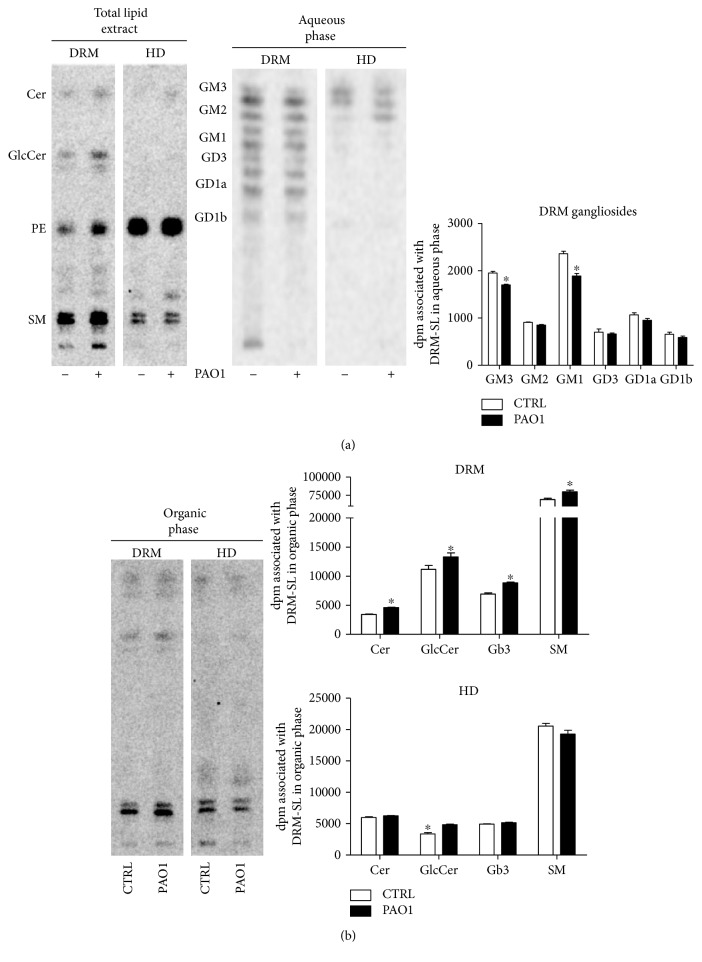
Sphingolipids composition of DRM and HD fractions obtained from CuFi-1 cells, infected or not with *P. aeruginosa*. (a) HPTLC of radioactive lipids contained in the total lipid extract, and aqueous phase obtained from detergent-resistant membrane (DRM) and high-density (HD) fraction of CuFi-1 cells, infected or not with PAO-1 (left). Semiquantitative graph of ganglioside composition of DRM from both infected and noninfected CuFi-1 cells. Data are expressed as radioactivity associated with the aqueous phase (right). (b) HPTLC of radioactive lipids contained in the alkali-treated organic phase obtained from the detergent-resistant membrane (DRM) and high-density (HD) fraction of CuFi-1 cells, infected or not with PAO-1 (left). Semiquantitative graph of sphingolipid species contained in the organic phase of DRM and HD fraction obtained from both infected and noninfected CuFi-1 cells. Data are expressed as radioactivity associated with the organic phase. The same amount of fraction were loaded on HPTLC. Cer: ceramide; GlcCer: glucosylceramide; PE: phosphatidylethanolamine; Gb3: globotriaosylceramide; SM: sphingomyelin. ^∗^*p* < 0.05 versus CTRL.

As a further step, we analyzed the SL composition of DRM and HD of CuFi-1 cells, infected or not with PAO1. Interestingly, we found a slightly decreased radioactivity in the aqueous phase derived from DRM and HD fraction of infected cells compared to noninfected cells (Figure 12(a)).

The lower radioactivity in the aqueous phase derived from DRM of infected cells could be attributed to gangliosides GM3 and GM1 (Figure 12(a)). The analysis of nonpolar SLs of DRM fraction of CuFi-1 cells infected with PAO1 showed a 22–27% increase of ceramide and a slight increase of Gb3, SM, and GlcCer (Figure 12(b)). The organic phase of HD fraction of CuFi-1-infected cells is only characterized by a modest increased GlcCer content compared to noninfected cells (Figure 12(b)).

**Figure 13:**
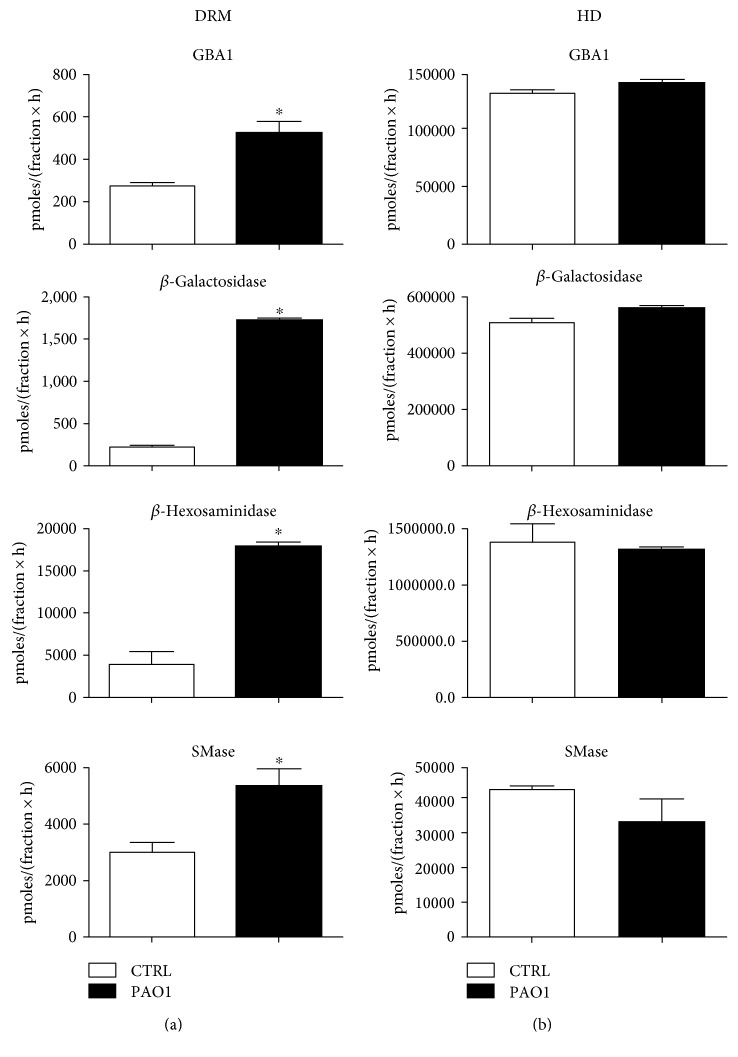
Effect of *P. aeruginosa* infection on hydrolases activity associates with DRM and HD fractions. The measurements of the hydrolases activity were conducted on DRM (a) and HD (b) fraction obtained from CuFi-1 cells infected or not with PAO-1, using an in vitro assay based on artificial fluorigenic substrates. The enzymatic activity was evaluated on the same volume of fraction and expressed as pmoles of product/total volume of fraction per hour. ^∗^*p* < 0.0001 versus CTRL.

Considering the differences in the SL pattern of gradient fractions, especially of DRM of infected and noninfected cells, we hence investigated the effect of infection on hydrolases in DRM fraction. Our results show significantly increased activities of DRM upon infection with PAO1 (Figure 13).

This data demonstrates that GBA1 and SMase activities are more than doubly increased in DRM derived from infected cells, whilst *β*-Gal and *β*-Hex increase over 3-fold. We measured the same hydrolases activity in HD fraction, but we did not find a significant difference in enzymatic activity of infected and noninfected cells (Figure 13).

## 4. Discussion

Several studies in different CF models strongly support the existence of a relationship between SLs, inflammatory response, and susceptibility to bacterial infection. In particular, increased PM Cer levels in CF bronchi seem to be correlated with the proinflammatory status characterizing CF lung disease [14].

In accordance with previous studies, we found that CuFi-1 and NuLi-1 cells are characterized by a SL composition recalling the features found in lung tissue of CF and non-CF patients [14]. An important difference was observed in ganglioside composition between CuFi-1 and NuLi-1 cells. The main ganglioside of CuFi-1 cells is GM3, whereas GM1 predominates in NuLi-1 cells. The differences in ganglioside composition between CF and not-CF cell lines are in line with evidence reported by Itokazu and colleagues [41], who described a direct correlation between ganglioside GM1 levels at PM and CFTR expression. In addition, as observed in CF lung tissue [11], CuFi-1 cells are characterized by a high content of Cer.

The activity of the PM glycohydrolases is another important difference between these two cell lines, since CuFi-1 cells are seemingly being characterized by higher activity of PM glycohydrolases compared to NuLi-1 cells.

Hydrolysis of the SLs directly triggered at PM by PM hydrolases may play an important role in regulating several physiological and pathological cellular processes [23].

Interestingly, part of Cer in bronchial epithelial cells is produced in response to *P. aeruginosa* infection through SM catabolism at PM by the acid-sphingomyelinase [42]. Conversely, bioactive Cer can be produced by action of PM glycohydrolases through GSL degradation at the cell surface, as shown in human fibroblasts [35, 43] and cancer cells [44]. However, limited information is available on the involvement of these enzymes in CF lung disease. Among PM glycohydrolases, nonlysosomal *β*-glucosylceramidase GBA2 plays a crucial role in the inflammatory response in CF bronchial epithelial cells.

GBA2 is an important player in the regulation of SL homeostasis at PM and is also a key enzyme in production of Cer at this site. Interestingly, we found that the main difference in PM activities between CuFi-1 and NuLi-1 cells is attributable to GBA2, which was 4-fold higher at the cell surface of CF bronchial epithelial cells.

Our recent data showed that pharmacological inhibition of GBA2, along with downregulation of its expression, is associated with significant reduction of inflammatory response to *P. aeruginosa* infection. Moreover, knocking down of GBA2 reduced the intrinsic proinflammatory state of CF epithelial bronchial cells [25].

To further support the role of GBA2 in CF inflammation, we overexpressed this enzyme tagged with GFP at C-terminal in CuFi-1 cells. GBA2-overexpressing cells were characterized by a strong increase of PM *β*-gal, which is suggestive of the existence of a crosstalk among different catabolic enzymes.

As expected, GBA2-overexpressing cells were characterized by a different SL profile, characterized by a marked decrease of GSL (e.g., GM3, Gb3, and GlcCer), followed by increased Cer and SM content. The lower increase of Cer level may be due to the formation of a complex between GBA2 and sphingomyelin synthase 2, which converts Cer to SM, as earlier suggested [39].

Since GBA2 is present within two different cellular compartments (i.e., PM and ER-Golgi), data on its effects on the SL pattern allow postulating two different hypothesis:

1. The increased PM-GBA2 activity, along with *β*-Gal, induces a catabolic unbalance which is not offset by de novo biosynthesis of complex GSL.
2. The increased GBA2 activity in ER/Golgi reduces GlcCer values, thus decreasing substrate availability for de novo synthesis of GSL complex.

These speculations need further studies that we plan to address in the near future.

As regards the inflammatory state of CuFi-1 cells overexpressing GBA2, an increased IL-8 secretion was found to be associated with overexpression of GBA2. These results support the role of GBA2 in the activation of inflammatory response in CF. However, GBA2 is not the only SL-hydrolytic enzyme at the cell surface. Several enzymes involved in catabolism of complex GSL at PM have been identified, including GBA1, *β*-Gal, *β*-Hex, sialidase Neu3, and SMase. In particular, *β*-Hex and *β*-Gal were found in the lipid rafts of Jurkat T-lymphocytes, where they are seemingly involved in local reorganization of these membrane areas. The involvement of this machinery has never been described so far in inflammatory response of CF.

The first evidence that we obtained was a clear effect of bacterial infection on DRM organization, since the DRM of infected cells was associated with increased SL content compared to uninfected cells.

The SL composition of DRM in infected cells is characterized by a reduction of GSL, combined with an increase in simple SL, including Cer.

This is evocative of a local activation of catabolic machinery. Interestingly, the activities of *β*-Gal, GBA1, *β*-Hex, and SMase were found to be increased in DRM isolated from infected cells.

**Figure 14:**
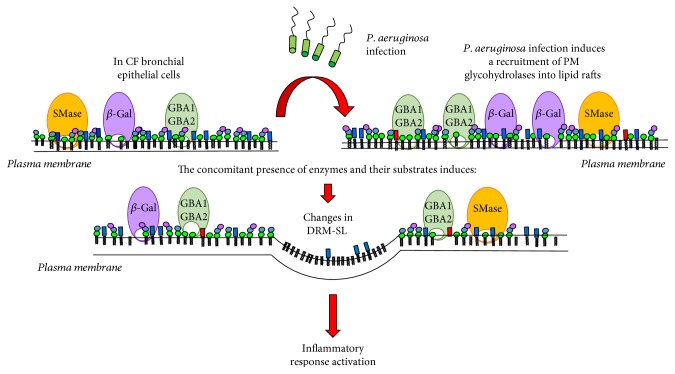
Graphical representation of the effect of *P. aeruginosa* infection on CF bronchial epithelial cells.

Taken together, these results suggest that *P. aeruginosa* infection causes an important rearrangement of the PM structure and composition in CF cells (Figure 14). This bacterial infection would probably lead to recruitment of glycohydrolases in specific membrane domains, enriched in SLs. The concomitant presence of glycohydrolases and their substrates induces significant changes of SL composition, which together with Cer organize specific platforms involved in activation of inflammatory response. These findings open new challenges for investigating the signaling cascade triggered by modification of DRM structure in CF inflammatory response.

## 5. Conclusion

In conclusion, our data further support the role of GBA2 in inflammatory response occurring in CF bronchial epithelial cells. In addition, we highlight the role of GBA2 in the proinflammatory state occurring in CF bronchial epithelial cells. We demonstrated that *P. aeruginosa* infection in CF bronchial epithelial cells causes significant changes in SL and protein composition within specific membrane areas involved in cell signaling regulation.

The structural modification of these signaling platforms may be involved in regulating the inflammatory response in CF.

Finally, we believe that the identification of specific SL playing an active role in inflammatory response of CF cells could pave the way to future therapeutic strategies for treatment of inflammation in CF. However, further studies, comprising *in vivo* validation, will be necessary to unravel the involvement of the SL in the signaling cascade responsible for the regulation of inflammatory response in CF.
